# Design and characterization of small-diameter tissue-engineered blood vessels constructed by electrospun polyurethane-core and gelatin-shell coaxial fiber

**DOI:** 10.1080/21655979.2021.1969177

**Published:** 2021-09-14

**Authors:** Yuanguo Zhang, Yuhao Jiao, Cong Wang, Chengchao Zhang, Han Wang, Zengguo Feng, Yongquan Gu, Zhonggao Wang

**Affiliations:** a Department of Vascular Surgery, Xuan Wu Hospital of Capital Medical University, Beijing, China; b Division of Biomaterials, National Institiutes for Food and Drug Control, Beijing, China; c School of Materials Science and Engineering, Beijing Institute of Technology, Beijing, China

**Keywords:** Tissue-engineered vascular graft (TEVG), vascular remodeling, coaxial fiber, polyurethane (PU), gelatin, mechanical properties

## Abstract

Substitution or bypass is the most effective treatment for vascular occlusive diseases.

The demand for artificial blood vessels has seen an unprecedented rise due to the limited supply of autologous blood vessels. Tissue engineering is the best approach to provide artificial blood vessels. In this study, a new type of small-diameter artificial blood vessel with good mechanical and biological properties was designed by using electrospinning coaxial fibers. Four groups of coaxial fibers vascular membranes having polyurethane/gelatin core-shell structure were cross-linked by the EDC-NHS system and characterized. The core-shell structure of the coaxial vascular fibers was observed by transmission electron microscope. After the crosslinking, the stress and elastic modulus increased and the elongation decreased, burst pressure of 0.11 group reached the maximum (2844.55 ± 272.65 mmHg) after cross-linking, which acted as the experimental group. Masson staining identified blue-stained ring or elliptical gelatin ingredients in the vascular wall. The cell number in the vascular wall of the coaxial group was found in muscle embedding experiment significantly higher than that of the non-coaxial group at all time points(p < 0.001). Our results showed that the coaxial vascular graft with the ratio of 0.2:0.11 had better mechanical properties (burst pressure reached 2844.55 ± 272.65 mmHg); Meanwhile its biological properties were also outstanding, which was beneficial to cell entry and offered good vascular remodeling performance.

Polyurethane (PU); Gelatin (Gel); Polycaprolactone (PCL); polylactic acid (PLA);1-(3-Dimethylaminopropyl)-3-ethylcarbodiimide hydrochloride (EDC); N-Hydroxy succinimide (NHS); 4-Morpholine-ethane-sulfonic (MES); phosphate buffered saline (PBS); fetal calf serum (FCS); Minimum Essential Medium (MEM); Dimethyl sulfoxide (DMSO); hematoxylin-eosin (HE).

## 1. Introduction

Globally, the incidence of atherosclerotic disease is exceedingly high, [1] which may cause stenosis and occlusion of the large and small arteries of the whole body, [2,3] and is associated with cardiovascular and cerebrovascular diseases and peripheral vascular diseases. The outcome is severe. It could be life-threatening or deteriorate the life quality of the patients. [4] Vascular replacement or bypass surgery has been proved to be an effective approach for managing cardiovascular diseases. [5,6] Autologous blood vessels are currently the most ideal vascular substitutes. [7] However, it is difficult to obtain suitable blood vessels in clinical practice as atherosclerotic diseases usually attack multiple vessels in the body. To address this, exogenous vascular grafts include allogeneic vessels and artificial vessels have been pursued. Allogeneic grafts have limited clinical application due to the storage difficulty and its immune reaction. [8] Artificial blood vessels have been successfully used in the replacement of large diameter blood vessels (>6 mm) while small-diameter artificial blood vessels (≤6 mm) showed a low patency rate, and their clinical application results were unsatisfactory. [9] Therefore, it is imperative to develop ways to make new small-caliber artificial blood vessels to meet the huge clinical needs.

Nanofiber membranes produced by applying electrospinning technique have the good processing reliability and operability [10]. The electrospun blood vessels have a porous structure which potentially increases the adhesion of endothelial cells [11]. Electrospinning vascular graft often undergo thrombosis and restenosis, which will occlude the arterial lumen. [12] To address the problem of thrombosis and restenosis, vascular implants are usually coated with biomaterials. Natural biomaterials could enhance the adhesion of endothelial cells, thus rapid endothelialization has anticoagulatory effects and prevents thrombosis. Natural biomaterials have good biocompatibility, no immune rejection, and able to realize controlled slow-release and rapid degradation, but their mechanical properties are poor [13]. Comparatively, synthetic polyester materials have good mechanical properties, no immunogenicity, and strong plasticity, but perform more poorly in terms of biological affinity. [14,15] Blending the two materials to producing nanofibers may be an alternative to achieve better biocompatibility as well as mechanical properties. [16] At the single-fiber level, reports on the coaxial fiber having a core-shell structure formed by two types of materials and spun into small-diameter artificial blood vessels are scanty, because the proper blending ratio is usually difficult to achieve. [17]

Polyurethane (PU) as one type of synthetic polyester materials possesses excellent mechanical properties including high elongation at break and mechanical strength. [18] Its disadvantage lies in its poor biological properties, such as strong hydrophobicity and poor cell adhesion. Therefore, it’s often modified with natural biomaterials to make it favorable for soft tissue engineering applications. [19] Gelatin (Gel) is the main product of collagen after mild irreversible rupture and is a derivative of collagen, [20] which could improve biocompatibility and biodegradability of the fabricated scaffolds. Compared to natural biomolecules such as collagen and chitosan, gelatin is low-cost and slightly antigenic. [21,22] In addition to the above advantages, gelatin coating can also promote in situ endothelialization of electrospun vascular grafts. [23]

To sum up, this study attempts to design and fabricate coaxially-structured fibers with upstanding mechanical and biological properties, which could combine the advantages of polyester materials and natural biological materials. PU was utilized as inner core to provide mechanical support, while biodegradable natural biomaterial gelatin acted as a shell to provide a surface for cell adhesion. To find the optimal core-shell ratio, four groups of artificial blood vessels with different polyurethane/gelatin ratios were designed. The GE shell was stabilized by crosslinking. Vascular graft was characterized for its morphology, hygroscopicity, and mechanical properties. The cytotoxicity test and embedding experiment were further carried out to evaluate the biocompatibility, cell affinity and remodeling.

## 2. Materials and methods

### 2.1 Design and manufacture of electrospinning vascular stents

The tubular scaffolds were fabricated through electrospinning with polycarbonate polyurethane elastomer (TPUPC-3575A) as the raw material (Lubrizol, USA). Hexafluoroisopropanol at a concentration of 15% (w/v) was used as a solvent for Gel (shell) and PU (core). We dissolved the two ingredients in HFIP for at least 10 h under continuous agitation at room temperature.The optimal process parameters (voltage, flow rate, and needle-collector distance) were determined based on the fiber morphology and stability of the electrostatic jet in the electrospinning process. The injection velocity of PU and Gel was set according to the ratio of concentric rings. The positive and negative voltage in spinning was 18.55 kV/-2.85 kV at a temperature of 20°C, 14% humidity, and a spindle speed of 20rpm. The receive distance was 23 cm. A 16-gauge needle was used as the outer tube of the coaxial needle, with an outer diameter of 1.66 mm and an inner diameter of 1.26 mm. The inner tube was a 22-gauge needle with an inner diameter of 0.4 mm and an outer diameter of 0.7 mm. Two syringe pumps (Fusion 100) were used to fill the internal/external polymer feed solution into a 10 ml plastic syringe (Zhiyu Medical, Jiangsu, China). [24]

The parameters of each group were adjusted to achieve a stable process and appropriate mechanical properties. The injection speed and spinning time of each group are shown in Table 1; the thickness of the tube wall was maintained within 0.5 ± 0. 1 mm. The electrospinning process was performed in temperature and humidity-controlled electrospinning equipment (SS series, Yongkang Leye, China).

**Table 1.** Injection speed and spinning time of four groups of blood vessels in electrospinning

Groups	PU injection speed (mm/min)	Gel injection speed (mm/min)	Spinning time (D = 6 mm)
PU	0.2	0	80–90 min
0.11	0.2	0.11	55–60 min
0.25	0.2	0.25	40–45 min
0.35	0.2	0.35	35–40 min

### 2.2 Cross-linked with EDC/NHS

Gelatin is water-soluble, which needs to be cross-linked to maintain the stability of the coaxial fiber structure. Although the polyurethane material did not need to be crosslinked, the control and experimental groups were treated the same for the sake of homogeneity of the experiment 270 mg EDC (Sigma) and 167.5 mg NHS (Sigma) were successively added to 100 ml MES (30 mmol/L, sigma) and mixed in an ice bath for 2 h. [25] Then artificial blood vessels were added to the solution for cross-linked for 48 h. After cross-linked, the scaffold was oscillated in PBS 5 times at 37°C for 5 min each time.

### 2.3 Transmission electron microscopy (TEM)

We verified the prepared fiber was a complete core-shell structure, using micrographs from a transmission electron microscope (TEM JEOL JEM 1200EX, Japan). During the vascular spinning process in each group, non-conductive wood tweezers were used to clamp the copper mesh, and the fibers emitted from the coaxial nozzle were collected randomly. Next, the nanofibers were collected on the carbon-coated copper mesh and dried sufficiently to prepare samples for TEM examination.

### 2.4 Morphological examination of vascular membranes and measurement of fiber diameter

The four groups of the artificial vascular membranes were dried for 12 h in a vacuum oven and cut into 0.5 cm×0.5 cm square samples. The inner surface of vascular membrane was pasted on the copper table of the scanning electron microscope with conductive adhesive, then the sample surface was sprayed with gold. The surface morphology and diameter of the electrospun fibers were examined using Quanta200 field emission scanning electron microscope at 10kV acceleration voltage, and micrographs were taken. Based on the PESEM images, the fiber diameter was measured using Image-Pro Plus software. The diameter of 80 fibers was randomly measured according to FESEM micrographs with a scale of 50 μm.

### 2.5 In vitro cytotoxicity test

HFIP have potent cytotoxicity, and because they were used in the fabrication of blood vessels, we performed in vitro cytotoxicity test according to ISO 10993.5: 2009.

a) Sample Preparation: Blood vessels were cut into 0.5 × 0.5 cm membranes, disinfected with 75% alcohol, and then rinsed three times with a complete MEM medium (supplemented with 10% FCS).

b) Test Solution Preparation: The extract solution was prepared using prepared membranes with 1 ml complete MEM medium at 37°C for 24 h. MEM medium acted as the blank control. High-density polyethylene (Hatano Research Institute, Japan) and MEM medium containing 10% DMSO were used as the negative control and positive control, respectively.

c) Cotoxicity test: The L-929 cells (National Institutes for Food and Drug Control, China) was digested from the culture flask and the cell concentration was adjusted to 1 × 105/ml. [26] Exactly100ul cell suspension was seeded into the testing wells of the 96-well plates and cultured for 24 h to form a semi-confluent monolayer. The cells were exposed to 100ul test solutions for 24 h. Finally, 20ul CCK-8 was added and measurement of the optical density was taken at the wavelength of 450 nm.

d) Data analysis: Viab. % = 100× OD450e/OD450b. Here the OD450e is the mean value of the measured optical density of the test samples and OD450b is the mean value of the measured optical density of the blanks.

### 2.6 Coomassie Bright Blue staining and Bradford assay

After crosslinking, four groups blood vessel samples were cut into membranes with the size of about 0.5 × 0.5 cm. Prepared membranes were placed in a 24-well plate, dropped into 2–3 ml Coomassie bright blue G250 (Service Bio, China) for 5 min; Then the membranes washed with PBS 3 times for 5 min each time. The membranes were photographed and observed.

The Bradford assay is a sensitive method for measuring the concentrations of proteins. It is based on the shift in absorbance maximum of Coomassie Brilliant Blue G-250 dye from 465 to 595 nm following binding to denatured proteins in solution. Generate a standard curve by plotting the average absorbance at 595 nm as a function of concentration of protein standard. Determine the amount of protein for a given volume of sample using the standard curve above. For detailed steps, see references. [27]

### 2.7 Mechanical characterization

The stress-strain tests were applied to evaluate the biomechanical properties of the four groups before and after cross-linking. Since graft material encounters forces like axial stress and circumferential stress during the systolic and diastolic cycles, mechanical tests were performed on both axial and circumferential stress.

#### 2.7.1 Axial (radial) stress

**Figure 1.**
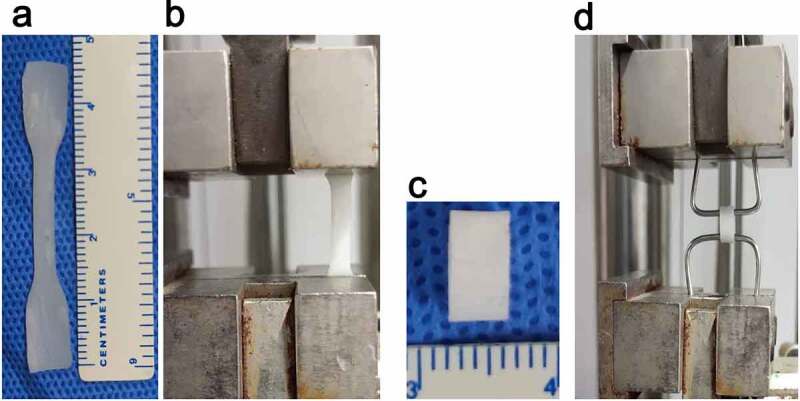
Axial and circumferential test samples and mechanical tests. (a) Dumbbell-shaped sample for the axial test (n = 5). (b) Axial mechanical test. (c) Annular sample for the circumferential test (n = 5). (d) Circumferential mechanical test

Radial stress: The sample was cut into a dumbbell shape with an effective area of 2 × 0.4 cm using a custom mold (Figure 1a). The sample thickness was measured using a digital micrometer. The sample was clamped with a uniaxial mechanical testing machine (DLL-5000, Shanghai, China) and stretched at a rate of 10 mm/min (Figure 1b) until it broke [28]. The fracture strength and fracture length were recorded during the experiment.

#### 2.7.2 Circumferential stress and bursting pressure

To study the circumferential mechanical properties of the artificial blood vessels, we performed the annular tensile test according to the method described by Laterreur et al. [29] and established the circumferential tensile strength and burst pressure.

**Figure 2.**
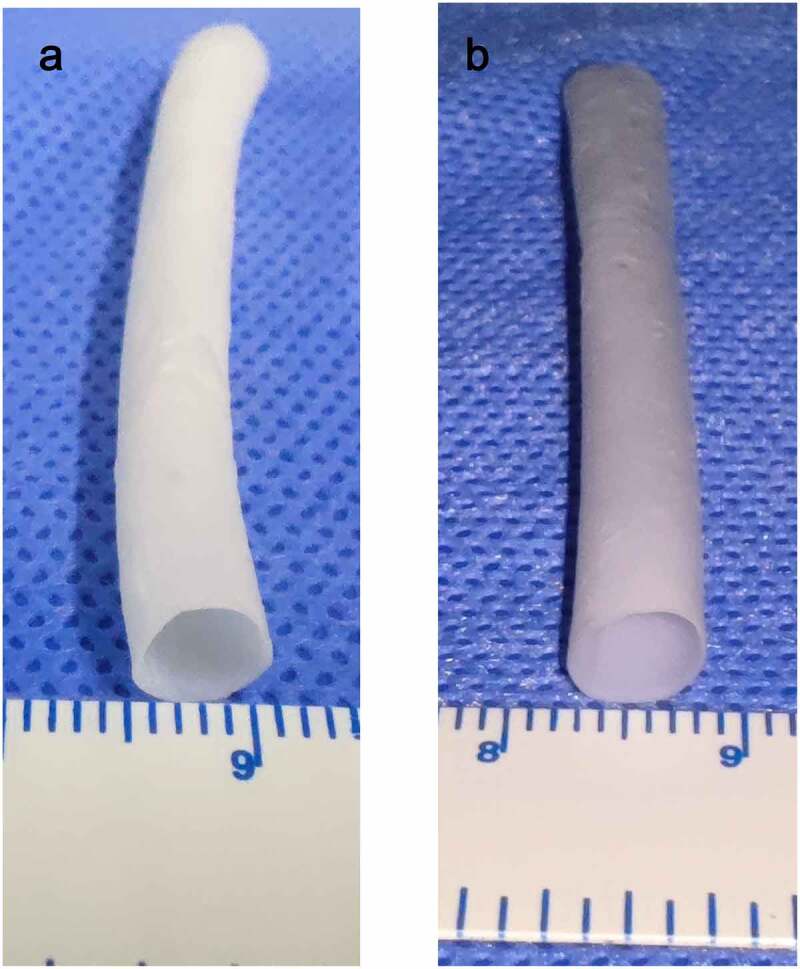
Electrospinning blood vessels with a diameter of 6 mm. A (PU group). B (coaxial group, represented by Group 0.11)

Briefly, the tubular scaffold was cut to a length of L0 = 6 mm (Figure 1c). The vascular segment was sheathed on a special pin measuring 1.5 mm in diameter (Figure 2d). The two pins were clamped in a uniaxial tensile test device (DLL-5000, Shanghai, China), and stretched for a distance at a speed of 5 mm /min until it was broken.

Based on the stress-strain curve (Figure 8a), the pressure Fb and elongation Sb at the time of fracture were recorded. The bursting pressure Pb was calculated as follows:

$$P_{b}=\frac{F_{b}\pi }{L_{0}d_{pin}\left(\pi +2\right)+2L_{0}s_{b}}$$

Where d_pin_ represents the diameter of the pin used in the ring tensile test, and L_0_ is the initial length. Lattereur et al. provided the derivation of this equation. [29]

### 2.8 Swelling rate

The water absorption was measured by weighing the sample in a dry and wet state, and the wet state means that the samples were soaked in PBS for 1 h. The increase in the relative weight over the dry weight was called the expansion rate. [30]

### 2.9 Histological analysis

The coaxial artificial blood vessels in group B were fixed in 4% paraformaldehyde for 48 h, embedded in paraffin, and cut into 5–10 μm slices. After dewaxing and rehydration, hematoxylin-eosin (HE) staining and Masson staining were performed. The composition and general morphology of the artificial blood vessels were assessed via HE staining. Masson trichrome stain was used to identify blue-dyed collagen fibers using a light microscope (IX71, Olympus, Japan).

### 2.10 Subcutaneous embedding experiment

The experimental rats were adult Wistar rats (320–350 g, 5 rats in each group). The experimental procedures were approved by the Animal Experiments Ethics Committee of Xuanwu Hospital of Capital Medical University. During the experiment, all rats received humane care in accordance with the Declaration of Helsinki of the World Medical Association. The rats were anesthetized with 1.5% isoflurane inhalation, after which their backs were shaved and disinfected with iodine volt. Vascular membranes (7 × 5 × 0.4 mm3, length/width/thickness) of group A and group B were implanted into the back muscles of rats. The wound was sutured with 3–0 monofilament nylon sutures. Samples were collected 1, 2, and 4 weeks after implantation. After paraffin embedding, HE staining was performed, and the number of nuclei in the middle 1/3 of the thickness of the membranes were calculated.

### 2.11 Statistical analysis

SPSS (IBM, Armonk, NY, USA) was used for statistical analysis. Digital data were expressed as x± SD. The student’s T-test was applied for a single comparison between the two groups, whereas one-way ANOVA analysis was used for multiple comparisons. A bar chart was created using GraphPad Prism (GraphPad Software Company, USA). The stress-strain curve was drawn using Origin software. The cell count was completed by Image-J software.

## 3. Results

In order to produce small diameter artificial blood vessels that can meet both good mechanical properties and remodeling performance, four groups of artificial blood vessels with different polyurethane/gelatin ratios were designed in this experiment. Then the core-shell structure was observed by transmission electron microscopy, four groups vascular graft were cross-linked by EDC-NHS. Coomassie bright blue was used to detect the gelatin components, and cytotoxicity test was used to detect the vascular residual toxicity. The changes of four groups vascular mechanical properties before and after cross-linking were measured by axial and circumferential mechanical tests, the group with the maximum burst pressure was selected as the experimental group and Masson staining was performed. Finally, the muscle embedding experiment was conducted to detect the vascular remodeling in the coaxial group and non-coaxial group.

### 3.1 Fiber morphology and fiber diameter of electrospun blood vessels

After stirring for more than 10 h, PU and Gel with 15% (w/v) concentration can be completely dissolved in hexafluoroisopropanol without bubbles and precipitation formation, and the viscosity is moderate. According to the spinning parameters in this experiment, an artificial blood vessel measuring 8 cm in length, 0.5 ± 0.1 mm in thickness, and 6 mm in diameter was obtained. Among the four groups of blood vessels, the group with a Gel injection rate of 0 was regarded as the pure PU group (Figure 2a). The other three groups were regarded as the coaxial group. The vessels in the coaxial group were represented by group 0.11(Figure 2b).

**Figure 3.**
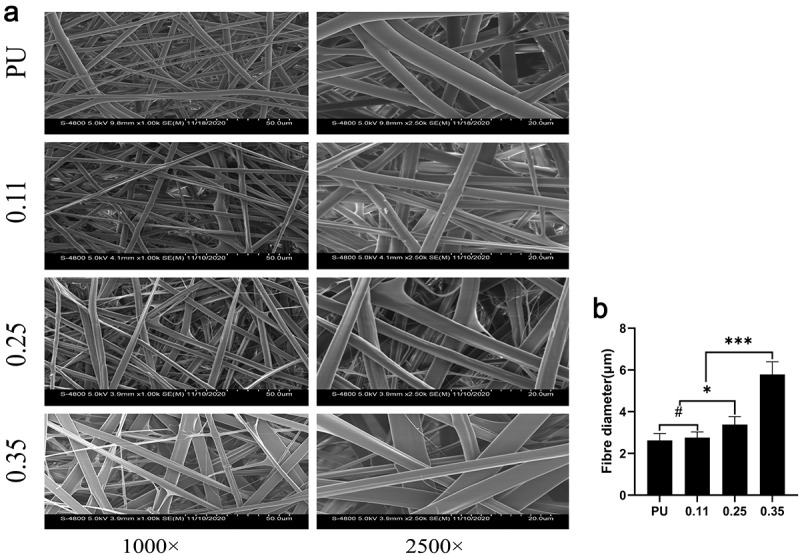
SEM results of four groups of artificial blood vessels. (a) Fiber morphology of the four groups of blood vessels. (b) Fiber diameters of the four groups of blood vessels. (# represents no statistical significance, * represents P < 0.5, ** represents P < 0.01, and *** represents P < 0.001, the same below)

**Figure 4.**
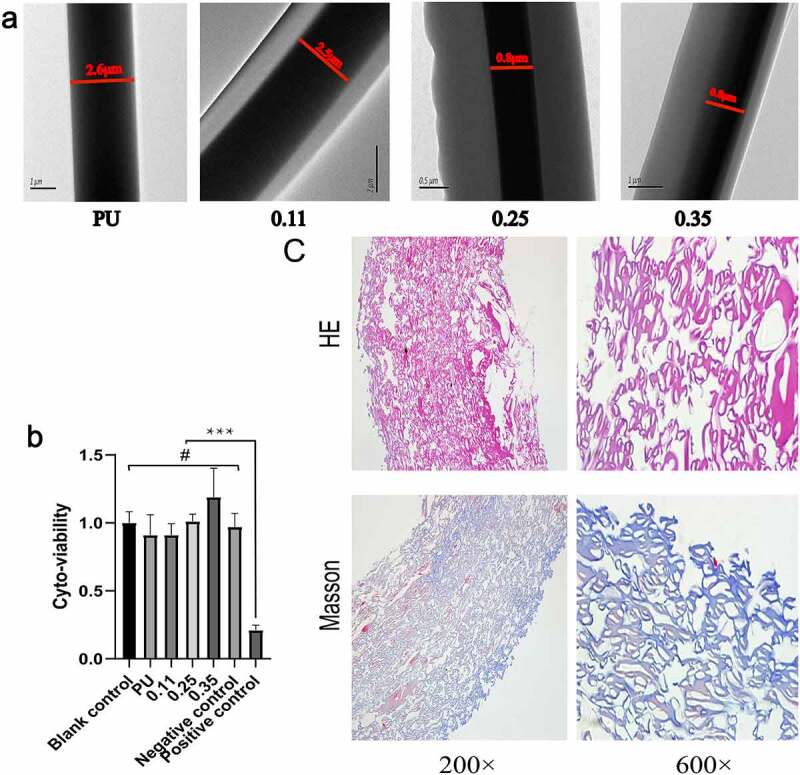
Results of transmission electron microscopy, cytotoxic test and Masson staining of blood vessel samples. Transmission electron microscopy results of four groups of vascular fibers (a). Cytotoxic test results of four groups of vascular membranes(b). Results of HE and Masson staining of blood vessels in the 0.11 group(c)

The average fiber diameter tended to get thicker with the increase of the feeding speed of shell solution when the injection speed of core polymer solution was 0.2 mm/min. However, there was no statistical difference in the fiber thickening in the 0.11 group (Figure 3b). Transmission electron microscopy revealed that, although the diameter of the whole fiber increased with the increase of gelatin injection speed, the diameter of the core polyurethane fiber decreased, such as group 0.35 and group 0.25 compared with group 0.11 (Figure 4a). The possible reason is that more Gel solution ejected per unit time squeezed the space of the nuclear layer fiber, and making its fiber thinner. In group 0.11, the thickness of the whole fiber was not statistically significant (Figure 3b) and the diameter of the core layer was not significantly thinner compared with the pure PU group (Figure 4a).

### 3.2 Transmission electron microscopy results

In the electrospinning process, single fiber samples from the four groups of blood vessels were randomly collected and noticeable core-shell structures were observed under a transmission electron microscope (Figure 4a). The inner darker region corresponds to the PU core layer while the outside lighter region to the Gel sheath. This is attributed to the different electron absorption and electron interactions between the semi-crystalline polyurethane and amorphous gelatin materials. In addition, in groups 0.25 and 0.35, in which the injection rate of shell Gel was faster, the thickness of the fiber shell was significantly thicker than that of group 0.11.

### 3.3 Coomassie Bright Blue (G250) staining results

**Figure 5.**
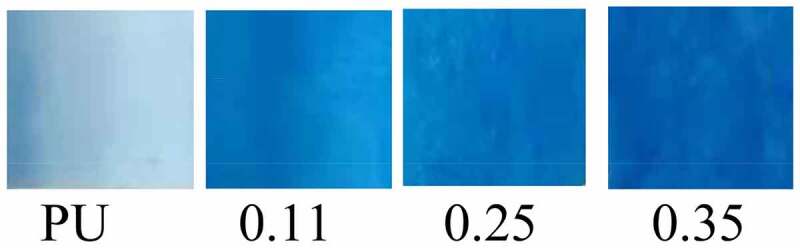
Results of Coomassie Bright Blue Staining of Four Groups of Vascular Membranes

Coomassie Bright Blue is highly sensitive to proteins and can rapidly blue (or cyan) the proteins that come into contact with it. Its light absorption value is proportional to protein content, therefore, Coomassie bright blue can be used for qualitative and quantitative analysis of protein [31]. Coomassie Bright Blue staining results showed that the blue staining of four groups vascular membranes became deeper and deeper with the increase of the injection velocity of gelatin (Figure 5). According to The Bradford assay protein quantitative analysis, the contents of vascular gelatin protein in four groups were calculated as follows: 0 mg/g (PU group), 26.57 mg/g (0.11 group), 41.36 mg/g (0.25 group), 68.85 mg/g (0.35 group). The results confirmed that the gelatin content increased with the increase of gelatin injection speed in the four groups vascular membranes.

### 3.4 In vitro *Cytotoxicity test results*

The cytotoxicity study on extracts from four groups vascular was performed to determine their residual toxicity. Compared with MEM medium as blank control and High-density polyethylene as negative controls, the cell viability of L929 cells exposed to the vascular membrane extracts from the four groups was not significantly reduced. With DMSO as the positive control, cell viability was significantly lower compared to other groups (Figure 4b). The cell activity of 0.35 group was higher than that of blank and negative control, possibly due to the high gelatin content, but the increase was not statistically significant(p > 0.05).

### 3.5 Mechanical characteristics

Studies have shown that the axial mechanical properties of atherosclerotic arteries are different from these of normal arteries, and the elastic modulus of atherosclerotic vessels is obviously larger [32]. Therefore, both axial and circumferential mechanics are equally important.

**Figure 6.**
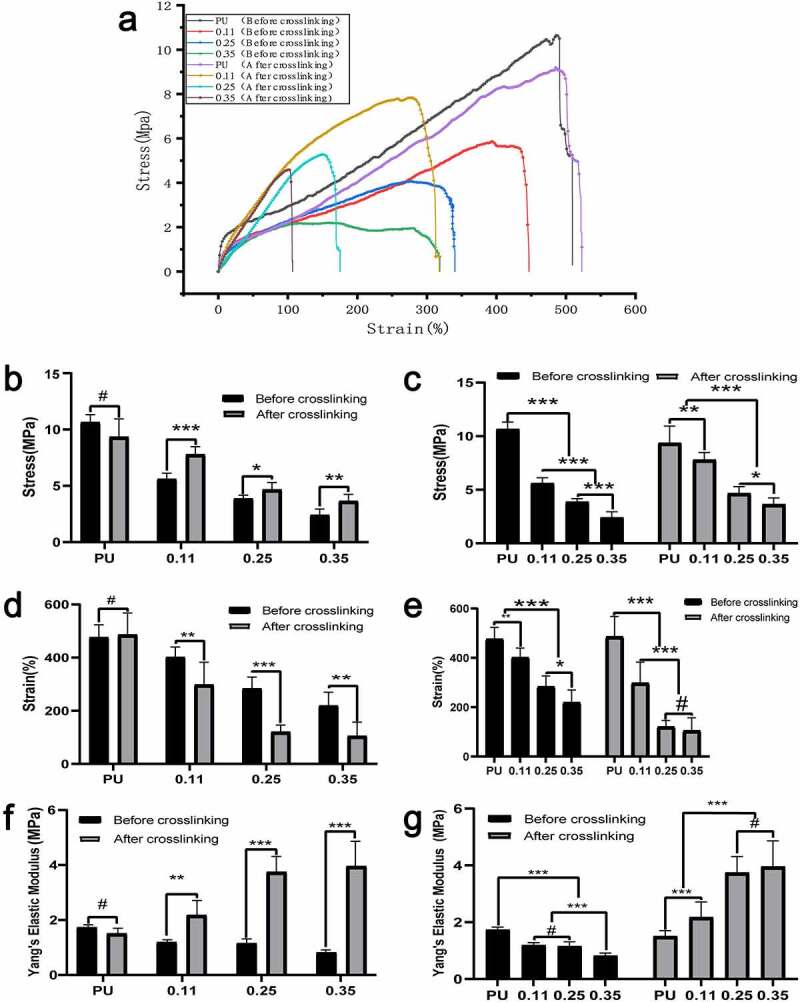
Axial mechanical test results of four groups of blood vessels before and after crosslinking. (a)Axial stress-strain curve of four groups of blood vessels before and after crosslinking. Intra-group comparison (b) and inter-group comparison (c) of maximum vascular stress in the four groups before and after cross-linking. Intra-group comparison (d) and inter-group comparison (e) of the maximum strain of vessels in the four groups before and after cross-linking. Intra-group comparison (f) and inter-group comparison (g) of vascular elastic modulus of the four groups before and after cross-linking

The four lines at the top of the axial stress-strain curve represent after cross-linking, and the four lines at the bottom represent before cross-linking (6ure 8A). From the right to the left, the gelatin content in the four groups gradually increased. The cross-linking had no significant effect on the axial mechanical characteristics of the pure PU group without gelatin, regardless of the maximum stress, maximum elongation, or elastic modulus (P < 0.01) (Figure 6b, Figure 6d, figure 6f, PU group).

The maximum axial stress and maximum elongation of the vessels in the three coaxial groups (both before and after cross-linking) decreased significantly with the increase of gelatin content. However, there was no statistical difference in the decrease of elongation in group 0.35 compared to group 0.25 after cross-linking (Figure 6c, Figure 6e, and Figure 6g). For the same group of vessels with the same gelatin ratio, the maximum stress after cross-connection increased significantly (Figure 6b), while the maximum elongation decreased significantly (Figure 6d). With the increase of gelatin content, the stress increases and the elongation decreases, indicating that the axial deformability of blood vessels decreases as the gelatin content increases. Notably, the cross-linking increased the axial elastic modulus of blood vessels significantly (figure 6f).

Moreover, the maximum stress, elongation, and modulus of elasticity decreased gradually with the increase of gelatin mass in the axial tension test before cross-linking (Figure 6c, Figure 6e, and Figure 6g). Possible reasons may be that, as the gelatin shell increases, it occupies some space in the core. Also, before bonding, the loose gelatin provides little mechanical support.

Axial tension detection after vascular cross-connection: With the increase of gelatin mass, the maximum stress and elongation decreased gradually. The decreasing amplitude of the maximum stress and elongation was smaller compared to that before cross-linking. The elastic modulus increased gradually (Figure 6c, Figure 6e, and Figure 6g). Possible reasons may be that, with the increase of gelatin shell, part of the core layer PU space is squeezed, which decreases stress. However, the cross-linked Gel can provide partial mechanical support, so the decreasing amplitude is smaller.

**Figure 7.**
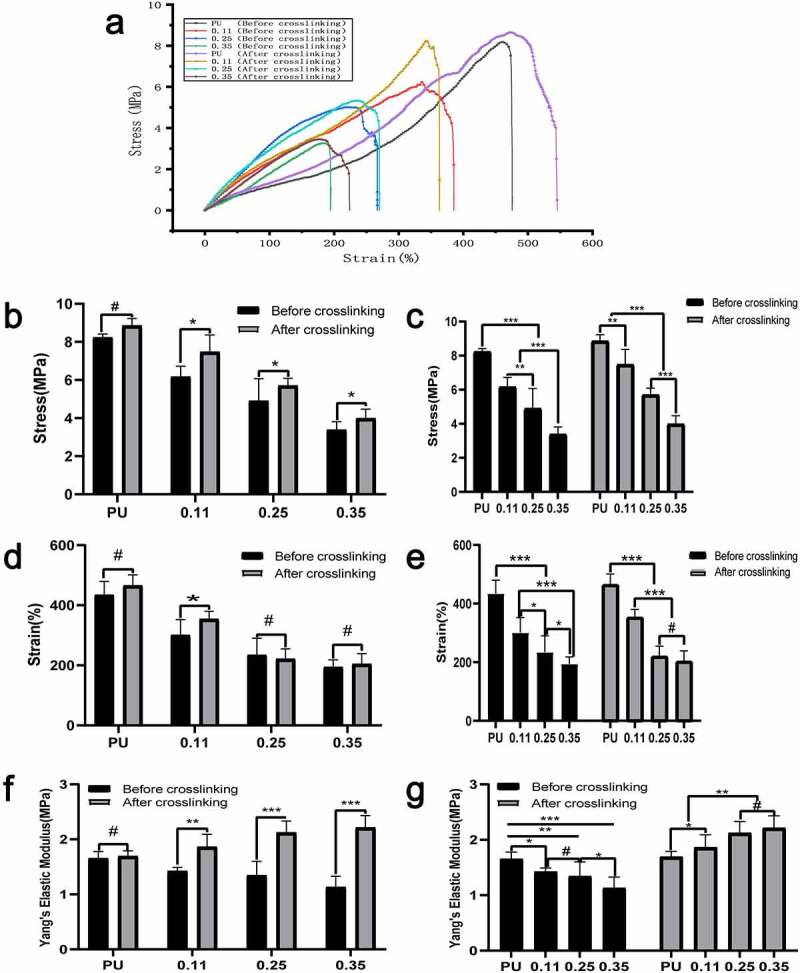
Circumferential mechanical test results of four groups of blood vessels before and after crosslinking. (a) Circumferential stress-strain curve of four groups of blood vessels before and after crosslinking. Intra-group comparison (b) and inter-group comparison (c) of maximum vascular stress in the four groups before and after cross-linking. Intra-group comparison (d) and inter-group comparison (e) of the maximum strain of vessels in the four groups before and after cross-linking. Intra-group comparison (f) and inter-group comparison (g) of vascular elastic modulus of the four groups before and after cross-linking

Compared with the axial stress-strain curve (Figure 6a), the influence of gelatin content and crosslinker on the circumferential mechanical characteristics is a little less. From the right to the left, the content of gelatin in the vessels of the four groups gradually increased. The cross-linking had no significant effect on the circumferential vascular mechanical characteristics of the pure PU group without gelatin (P < 0.01) (Figure 7b, Figure 7d, and figure 7f, the PU group).

Furthermore, the maximum circumferential stress and maximum elongation of vessels in the three coaxial groups containing gelatin (both before and after cross-linking) decreased significantly with the increase of gelatin content. However, there was no statistical difference in the decrease of elongation in group 0.35 compared to group 0.25 after cross-linking (Figure 7c, Figure 7e, and Figure 7g). For the same group of vessels with the same gelatin ratio, the stress after cross-connection increased significantly (Figure 7b), whereas the elongation decreased significantly (Figure 7d). With the increase of gelatin quantity, the stress increases and the elongation decreases, indicating that the circumferential deformability of blood vessels also decreases as the gelatin content increases. The cross-linking agent significantly increased the axial elastic modulus of blood vessels (figure 7f).

In the circumferential tension test before cross-linking, the maximum stress, elongation, and elastic modulus decreased gradually with the increase of gelatin mass (Figure 7c, Figure 7e, Figure 7g). But the decreasing amplitude was less than the axial stress and elongation (presumed to be attributed to the axial rotation of the receiving rod). Possible reasons may be that, with the increase of gelatin, part of the space of the core is squeezed; before the cross-linking, the loose gelatin provides little mechanical support, so the maximum stress and elongation decreases significantly.

In the circumferential tension test after cross-linking, the maximum stress and elongation decreased with the increase of gelatin (Figure 7c and Figure 7e). The possible reason may be that the gelatin occupied the space in the part of the core, and decreased the stress) but with the following characteristics:(a) Compared with before crosslinking, the decreasing amplitude of maximum stress and elongation of vascular samples is smaller, the reason is that gelatin after crosslinking can provide partial mechanical support, so the decreasing amplitude is smaller. And the elastic modulus increased gradually after cross-linking while elastic modulus decreased gradually before cross-linking (Figure 7g). (b) Compared to the maximum axial stress, with the increase of gelatin, the decreasing amplitude of circumferential stress is less than that of axial stress.

Considering that this may be due to the rotation of the receiving rod, the fibers are arranged along the circumferential direction rather than the axial direction, which leads to the number of fibers with circumferential stress at the same time is significantly more than that with axial stress, and then the change amplitude of circumferential stress decreases with the increase of gelatin.

**Figure 8.**
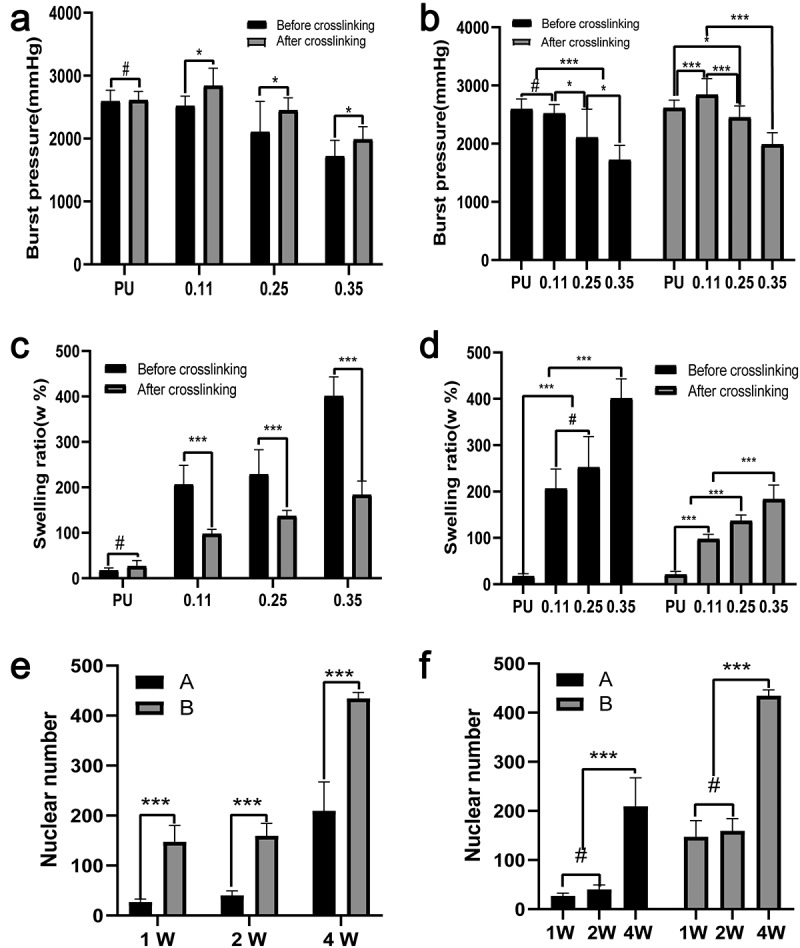
Statistical results of burst pressure, swelling rate and nuclear counts. Test results of burst pressure (a, b) and swelling rate (c, d) of four groups of blood vessels before and after cross-linking. Nuclear counts (e, f) in the middle part of the vascular membranes after 1, 2, and 4 weeks of animal embedding experiment in groups A and B. (A, C, E) Intra-group comparison. (B, D, F) Comparison between groups

The burst pressure was calculated according to the maximum stress, elongation at break, and other parameters of the circumferential stress-strain curve. The crosslinker had no obvious effect on the bursting pressure of the PU group, but the crosslinker could increase the bursting pressure of the coaxial groups significantly (Figure 8a). Both before and after cross-linking, the burst pressure decreased significantly with the increase of gelatin content (Figure 8b) but reached the maximum in group 0.11 after cross-linking. Possible reasons may be that in group 0.11, gelatin occupied the least core space, and the slight mechanical decline attributed to the core PU change was balanced best with the mechanical support provided by gelatin after crosslinking.

### 3.6 Expansion rate

The pure PU group exhibited strong hydrophobicity, so its water absorption is poor. Gelatin has good hydrophilic properties, and its hydroscopicity is obviously improved after the coaxial fiber is wrapped by gelatin. With the increase of gelatin content, the water absorption rate gradually increased. Except for the comparison between group 0.25 and group 0.11 before cross-linking, the increase of other groups showed a statistical difference (Figure 8c-d). After cross-linking, the water absorption of the vascular membrane decreased. For the four groups of blood vessels before crosslinking, the water absorption rate increased significantly but increased slightly after crosslinking (Figure 8d). For the same group of coaxial vessels with the same gelatin content, the crosslinker significantly reduced water absorption (Figure 8c).

### 3.7 Results of histological analysis

Collagen and gelatin fibrin in tissue can be dyed blue by Masson staining. Referring to the coaxial vascular schematic diagram in Graphical Abstract, the shells of PU/Gel coaxial fibers in the tube wall were blue stained. Considering the different directions of each fiber, it is impossible for every fiber to be completely perpendicular to the across section plane as shown in the Graphical Abstract. Therefore, in the Masson staining, the shells layer of the coaxial fibers can be circular, elliptical, or double-track blue dyed (Figure 4c, Masson, 600×).

### 3.8 Animal embedding experiments

**(Count the number of cells in the area between the blue lines)**

**Figure 9.**
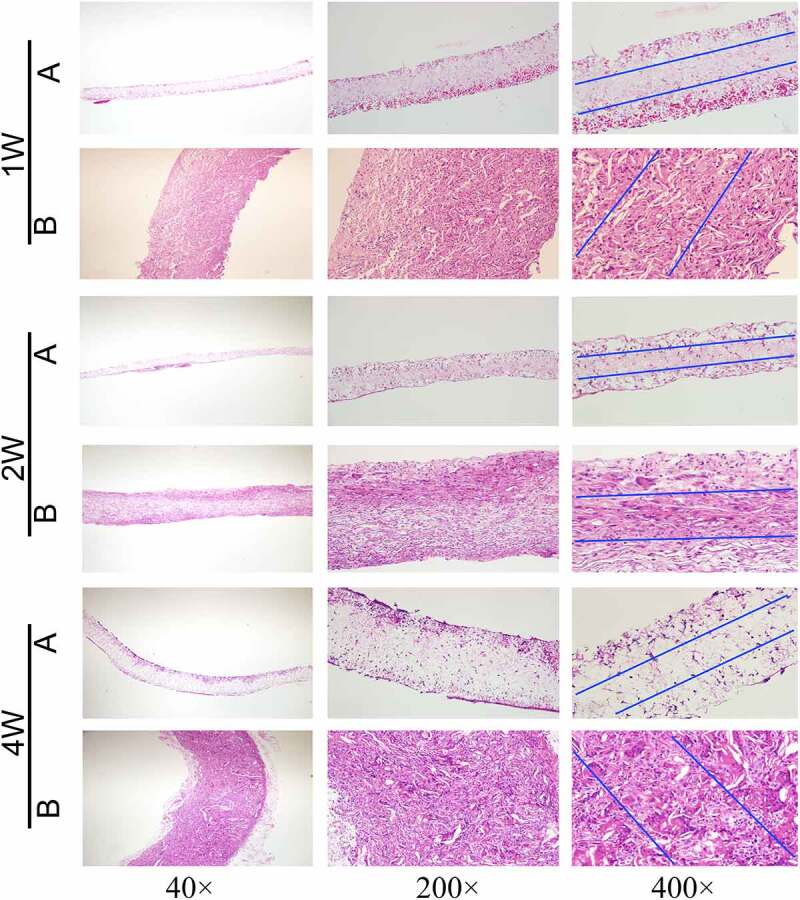
HE staining results of vascular membranes in group A and group B

Vascular membranes (n = 5) of group A and group B were embedded in the back muscles of rats, and samples were collected at 1, 2, and 4 weeks after implantation, respectively. The extracted vascular membranes were observed: The morphology of group A (pure PU group) showed no significant change compared with that before implantation, and it was slightly adherent to surrounding tissues. In group B (coaxial group), the vascular membrane was thickened with a rough surface and it severely adhered to the surrounding tissues. At low magnification (40×), HE staining revealed that the thickness of the membrane in group B was significantly higher than that in group A (Figure 9).

In addition, HE staining showed clear micrographs of the nucleus and general cell morphology. At high magnification (400 ×), only a few cells were present at the edge of the membrane in group A, while cells could be seen in the whole layer of the membrane in group B (Figure 9).

The number of nuclei can represent the number of cells. Five high-power fields were randomly selected for each group and the Image-J software used to count the number of cells within the middle 1/3 thickness of the membrane under high-power microscopy. The mean and standard deviation were calculated. At three time points, the number of cells in the middle part of the vascular membrane in group B (coaxial group) was significantly higher than that in the pure PU group (P < 0.001). At week 2, the number of cells in group A and group B did not increase significantly compared with week 1, and the number of cells in the two groups only increased significantly at week 4 (P < 0.001) (Figure 8e, f).

## 4. Discussion

Polyester nano/micro-fibers generally have good mechanical properties, though their biocompatibility may not be able to promote the cellular attachment and growth. [33] Although natural biological material possesses better biocompatibility than synthetic material, but their poor mechanical properties may restrict their use as artificial vascular grafts. [34] Co-axial electrospinning is an emerging manufacturing technology that can integrate the advantages of polyester materials and natural biological materials by producing core-shell fibers. [35] By using a co-axial needle during the electrospinning process and optimizing various parameters, core-shell nanofibers can be fabricated.

Ideally, artificial vascular graft degradation rate should be consistent with the rate of new tissue formation. [36] Based on this concept, an artificial vascular graft composed of semi-degradable coaxial fibers was designed in this study. The core fiber was made from polymer polyester materials, which could provide stable mechanical support, while the shell layer was made from natural biological materials with good biological properties, which could gradually degrade. Such nano/micro-fibers had good biocompatibility as well as good mechanical characteristics to achieve the requirement of a tissue-engineered vascular graft. [24]

The main role of the core layer is to provide mechanical support. The in vitro biological stability of PU was previously studied from the mechanical point of view under long-term oxidation treatment [37], which has superior mechanical properties to PCL. Collagen is one of the most important extracellular matrix proteins in the vascular system; Nevertheless, electrospinning using strong organic solvents usually induces collagen degeneration to gelatin [38]. Gelatin has good biodegradability and excellent biocompatibility and non-antigenicity, which are comparable to collagen. [39,40] Studies have shown that the arginine-glycine-aspartic acid sequence in its primary structure is the most important characteristic of gelatin as an effective macromolecule in tissue engineering. [21] In addition, gelatin is widely available and cheap. Therefore, polyurethane was selected as the core and gelatin as the shell, and four groups of different core-shell ratios were designed to manufacture artificial blood vessels.

Studies on small diameter artificial blood vessels spun by PU/Gel coaxial fibers are currently limited. In the paper by Detta et al., polyurethane fibers and gelatin fibers were cross-spun into a network and then prepared into artificial blood vessels. [41] In the paper by Wang et al., PU/Gel coaxial membrane was served as the outer wrapping of implanted sensors in vivo, rather than make artificial blood vessels. [42] It has also been reported that PU and Gel are simultaneously dissolved in the same solution for blending, which can promote the proliferation of endothelial cells and smooth muscle cells under certain mechanical properties. [43] Compelling evidence shows that the adhesion and proliferation of endothelial cells are increased proportionately with the gelatin content in scaffolds [44]. Although numerous studies have explored the artificial blood vessels with coaxial fibers of PCL-gelatin core-shell structure, the mechanical properties of such vessels are insufficient [26,30,45]. Another study spun a layer of pure PCL on the outer layer of the coaxial artificial blood vessel to enhance its mechanical properties, [46] but it was prone to cause vascular stratification.

In our experiment, the gelatin in the shell layer of the artificial vascular fibers could be degraded gradually. Chen et al. studied the degradation rate of gelatin in coaxial fibers with different ratios of PCL/Gel [47]. The fibers gradually became thinner with the degradation of the gelatin, and the pore size and porosity of the vessels increased significantly, which led to the accelerated graft remodeling via higher cellular infiltration and tissue deposition. [48] This is the biggest advantage of coaxial artificial blood vessel, and the key to achieving this aim is the appropriate ratio of polyurethane and gelatin. It can ensure not only sufficient mechanical support but also a certain thickness of the shell layer. Generally, in the polyurethane and gelatin blended system, the increase of gelatin content will reduce the maximum stress, maximum elongation, and make the blood vessels brittle. [49] However, the relative increase of PU can significantly improve the degradation resistance, maximum stress, and elongation of these scaffolds without any change in cell viability. [50] In the present experiment, to ensure the mechanical properties of the core PU, a constant injection rate of 0.2 mm/min was designed, and the injection velocity (mm/min) of the four groups of shell gelatin increased successively, 0, 0.11, 0.25, 0.35. It is expected to obtain the artificial blood vessel with a certain thickness of shell gelatin, when core diameter supplies sufficient mechanics.

The influencing factors of fiber diameter in electrospinning can be mainly summarized into three categories: [51,52] One is the properties of the spinning fluid, such as polymer concentration, viscosity, surface tension and electrical conductivity. The second involves working conditions of the spinning machine, such as the applied voltage, the fluid flow rate, the nanofiber-collected distance, and the nozzle diameter of spinneret. And the third is spinning environment, such as temperature and humidity. The fiber diameters and mechanical properties of some electrospinning coaxial fiber membranes are shown in Table 2. Since the spinning conditions are completely different in each study, the values in this table are only of reference value. In the references collected in Table 2, studies on the biological properties of coaxial fiber membranes were performed using endothelial cells and smooth muscle cells for cell proliferation assay in vitro. Cell proliferation within 1 week only confirmed the biocompatibility of the coaxial fiber materials. None of these papers has conducted in vivo experiments to study the remodeling properties of vascular membranes. [26,30,53,54]

**Table 2.** Mechanical properties of different electrospinning coaxial fiber membranes

Core material (w/v), *velocity*	Shell material (w/v), *velocity*	Fiber diameter (μm)	Maximum stress (MPa)	Maximum Strain (%)	Burst pressure (mmHg)	Ref.
**15%PU** *0.2 ml/min*	#	2.62 ± 0.34	8.6 ± 0.37	465.99 ± 35.21	2617 ± 130	A B
**15%PU** *0.2 ml/min*	**15%Gel** *0.11 ml/min*	2.76 ± 0.27	7.48 ± 0.39	354.36 ± 25.86	2845 ± 130	
**1%PU** *NA*	**5%Gel** *NA*	0.21 ± 0.07	8.5	40	540 ± 140	[30]
**1%PLA** *NA*	**5%Gel** *NA*	0.27 ± 0.09	3.0	120	393 ± 140	
**1%PCL** *NA*	**5%Gel** *NA*	0.33 ± 0.07	2.6	140	380 ± 180	
**20%PCL** *0.18 mm/min*	**10%collagen** *0.06 mm/min*	#	3.4 ± 0.04	159 ± 1	1568 ± 30	[26]
**6%PU** *0.8 mL/h*	**10%Gel** *1.2 mL/h*	1.15 ± 0.13	0.93 ± 0.14	133.29 ± 7.17	NA	[53]
**8%PU** *0.6 mL/h*	#	0.35 ± 0.09	2.3 ± 0.85	191.2 ± 61.1	NA	
**12%PU** *1.2 mL/h*	#	1.10 ± 0.21	0.97 ± 0.05	261.0 ± 12.09	NA	
**10%PU** *4 ml/h*	#	0.78 ± 0.16	21.3 ± 2.1	#	3326 ± 78	[54]

We compared the mechanical properties (burst pressure) of our electrospun scaffolds with the autologous grafts, which are today’s gold standard for vascular bypass surgeries. [55] And found the burst pressure range of the great saphenous vein was 1680–3900 mmHg, [56]and that of the internal mammary artery was 2000–3196 mmHg. [56,57] According to Konig et al., a minimum burst pressure for TEVG was 1700 mmHg. [57] We could therefore conclude that our artificial blood vessels have suitable mechanical properties and biological properties to serve as a vascular graft.

Polycaprolactone (PCL); polylactic acid (PLA); NA stands for missing in the original source; # indicates no value. The first and second rows of the table represent the control group (group A) and the experimental group (group B) in the experiment.

The experimental results confirmed that with the increase of gelatin content, both the maximum stress and elongation would decrease. Moreover, it can be found that although the core velocity remains unchanged, the acceleration of the shell injection velocity will squeeze the core space and make the diameter of the core fiber smaller. This effect was the least in group 0.11, and greater in groups 0.25 and 0.35. Different from our initial expectation, a certain core PU injection speed can provide a stable PU fiber diameter to ensure the mechanical properties of blood vessels. This new finding has not been reported in previous studies. To validate this point, a series of mechanical analyses were performed. The results showed that both the vascular maximum stress and elongation decreased with the increase of gelatin injection velocity at the same core layer injection velocity. The transmission electron microscopy of the four groups of fibers provided intuitive evidence.

Coomassian bright blue staining confirmed from a macroscopic perspective that there was no protein component in the vascular membrane of the PU group. With the increase of gelatin content in the coaxial group, the blue color deepened, which indirectly confirmed the existence and increased of gelatin. The results of quantitative analysis of gelatin Coomassie bright blue staining are as follows:0 mg/g (PU group), 26.57 mg/g (0.11 group), 41.36 mg/g (0.25 group), 68.85 mg/g (0.35 group), the increase of gelatin content in the four groups of vascular membranes was also confirmed. Masson staining was performed for the selected optimal proportion group (group 0.11). The results also revealed that the ring, elliptical, or double-track blue-dyed gelatin components were distributed in the pipe wall, which confirmed the existence of coaxial gelatin components from the microscopic perspective.

Rapid cell infiltration of the grafts is the first and crucial step for vascular remodeling. [58] In the embedding experiment, the cell number in the vascular wall of the coaxial group was significantly higher than that of the non-coaxial group at all time points(p < 0.001). The experimental results show that coaxial group vascular membrane was more conducive to cell entry and the remodeling. This result is consistent with our design philosophy, that is, the gelatin degradation in the shell provides space for the cells to entry.

There are still some limitations in our experiment. First, electrospinning does not guarantee every fiber in the tube wall a coaxial fiber. Secondly, no group with a lower proportion than group 0.11 was designed and the shell velocity of the new group should be between 0.68 and 0.11 for experiments, which may have better mechanical properties, and the thickness of shell layer will be sacrificed. In addition, subcutaneous embedding experiment results are insufficient. HE staining was used to estimate the number of nuclei. Immunohistochemical staining and qualitative analysis should have been performed in the next step to determine the type of cells in the membrane. Last but not least, no animal experiments were conducted to evaluate the efficacy and safety of vascular transplantation in vivo, which is necessary before clinical application. The next step in our research will address these limitations.

## 5. Conclusion

In this study, four groups of PU/Gel scaffold with core/ shell fiber structure were successfully prepared via coaxial electrospinning. EDC-NHS crosslinking was found to be able to increase vascular stress, elastic modulus, and reduce elongation. The increase of the injection velocity of gelatin reduced the diameter of the core fiber. Gelatin was found to decrease vascular maximum stress and elongation. Burst pressure of 0.11group reached the maximum (2844.55 ± 272.65 mmHg) after cross-linking. The cytotoxicity and embedding experiment further indicated that the scaffold samples had good biocompatibility and cell affinity, allowing the infiltration of cells into its interior. The PU/Gel scaffold with core/shell fiber structure prepared via coaxial electrospinning was thus proved to have a promising application in vascular tissue engineering.

## Highlights

1. Four groups of coaxial fibrous artificial blood vessels were electrospun.
2. Vascular mechanical properties and remodeling properties were studied.
3. EDC-NHS crosslinking could increase stress, and reduce elongation.
4. Burst pressure reached the maximum at 0.11 group after cross-linking.
5. Artificial vessels in coaxial group were beneficial to vascular remodeling.
